# Evidence-based Practice Interventions for Reducing Postoperative Pulmonary Complications: A Narrative Review

**DOI:** 10.2174/012210299X247199231128100613

**Published:** 2023-12-04

**Authors:** Gagandeep Dhillon, Venkata S. Buddhavarapu, Harpreet Grewal, Ripudaman Munjal, Ram Kishun Verma, Salim Surani, Rahul Kashyap

**Affiliations:** 1 Department of Internal Medicine. University of Maryland Baltimore Washington Medical Center, Glen Burnie, MD, USA; 2 Banner Baywood Medical Center, Banner Health, Mesa AZ, USA; 3 Radiology Associates of Florida, Pensacola, FL, USA; 4 Touro University College of Osteopathic Medicine, CA, USA; 5 Department of Sleep Medicine, Parkview Health System, Fort Wayne, IN, USA; 6 Department of Anesthesiology and Critical Care Medicine, Mayo Clinic, Rochester, MN, USA; 7 Texas A&M, College Station, TX, USA; 8 Department of Research, WellSpan Health, York, PA, USA

**Keywords:** Incentive spirometer, Inspiratory muscle training, Cough and deep breathing, Early mobilization, Postoperative pulmonary complications, Healthcare

## Abstract

**Background:**

Specific surgical procedures, such as upper abdominal and thoracic surgery, are connected to an increased predisposition of postoperative pulmonary complications (PPCs). The incidence of PPCs could vary approximately between 20-90% with upper abdominal surgery, which can be minimized by using treatment procedures that increase lung capacity and encourage inspiration. This review aims to examine the effectiveness of already existing evidence-based interventions that promote lung expansion, thereby preventing PPCs.

**Method:**

We mainly focused on the existing evidence of preoperative education on the incentive spirometer, early mobilization, directed coughing, deep breathing exercises, chest physiotherapy, and inspiratory muscle training (IMT) to prevent PPCs. The literature search was limited to experimental, observational studies, systemic reviews, and articles published in the last 15 years, January 2007- Dec. 2022, in PubMed and Google Scholar.

**Result:**

This initial search yielded a total of 5301 articles. All articles with titles not related to the topic were eliminated. 1050 records were screened, and the final review was conducted with 22 articles, including 13 randomized controlled trials (RCTs), four systemic reviews, one retrospective review, three observational studies, and one non-experimental study. Our review reveals mixed evidence for individual interventions, including but not limited to incentive spirometry, inspiratory muscle training, early mobilization, cough, deep breathing, *etc*. Some studies maintain that intervention is effective; others imply there is no substantial difference in the choice of intervention.

**Conclusion:**

The literature review concluded that patients who received multiple interventions showed significant improvement in pulmonary function postoperatively. However, definitive studies need to be conducted to solidify this conclusion.

## 1 INTRODUCTION

Postoperative pulmonary complications (PPCs) represent a significant issue for the healthcare system in the US. According to Kazaure **et al*.* [1], about 16.7% of patients undergoing surgery are affected by postoperative complications. Over 40% of these complications occur post-discharge. According to Shander **et al*.* [2], postoperative patients with PPCs have a 21% mortality rate at 30 days postoperatively compared to 2% for those without PPCs. Despite modern technology and advancements in anesthesia and surgery, PPCs remain the most common cause of increased morbidity and mortality [3].

PPCs are frequently a direct result of insufficient or inadequate lung expansion, prolonged shallow breathing, and being in the recumbent position. It is more common in postoperative abdominal patients because of a temporary dysfunctional diaphragm and the inability to clear mucous due to a decrease in the ability to cough. In addition, shallow breathing reduces breathing in the affected lung regions and may lead to atelectasis. These combined conditions put these patients at a greater risk for developing PPCs, which include pneumonia, atelectasis, lung fibrosis, tracheobronchial infection, and respiratory failure. Pulmonary atelectasis is the most common complication, and pneumonia is the leading cause of mortality [4]. Lasting anesthetic effects, incisional pain, and atelectasis may result in a lung infection as a secondary occurrence [5].

Many risk factors for developing PPCs can be detected when evaluating the patient and taking their history. Several measures, including early ambulation, have been used to prevent PPCs. Research shows innovative and thoughtful use of evidence-based therapeutic protocols can substantially improve lung compliance in postoperative patients.

According to Kazaure **et al*.* [1], more than 40% of PPCs happen after patients are discharged from the hospital. The risk factors include obesity, comorbidities, advanced age, smoking, and existing chronic lung disease [6]. The anesthetic factors include the type and duration of anesthesia and analgesics. The type and length of surgery and the degree of the surgical cut also play an important role [7]. Lying supine on a bed for a long time may contribute to complications. According to Duarte and Machado [8], measures aimed at ensuring proper initial assessment to detect comorbidities and address them appropriately, improving lung ventilation pre-, intra-, and post-surgery, and preventing infection through clean and sterile procedures can significantly reduce the risk of PPCs. For postoperative patients, does the use of evidence-based practice interventions, such as preoperative education on an incentive spirometer (IS), early mobilization, directed coughing, deep breathing exercises, chest physiotherapy, and inspiratory muscle training (IMT), reduce the risk of developing pulmonary complications during the postoperative period?

There is a lack of quality evidence supporting the current practice and guidelines for surgical units in preventing PPCs. However, it is critical to identify best practices to provide postoperative patients with effective and efficient care in preventing PPCs. Improving lung ventilation and clearing can significantly reduce the occurrence of PPCs. We aimed to conduct a thorough review of PPCs among surgery patients.

## 2 MATERIALS AND METHODS

The literature search was limited to experimental, observational studies, systemic reviews, and articles published in the last 15 years, January 2007- Dec. 2022. The search was performed using the help of the following databases: PubMed and Google Scholar. Only studies with participants who had undergone surgery were included in the review to evaluate the effectiveness of each intervention. The initial search terms included were “postoperative pulmonary complications prevention,” “cough and deep breathing exercises,” “early mobilization,” “incentive spirometer,” and “inspiratory muscle training.” This initial search yielded a total of 5301 articles. All articles with titles not related to the topic were eliminated. 1050 records were screened, and the final review was conducted with 22 articles, including 13 randomized controlled trials (RCTs), four systemic reviews, one retrospective review, three observational studies, and one non-experimental study (Fig .**1**). The interventions were preoperative IS education, coughing and deep breathing exercises, early mobilization, and inspiratory muscle training.

## 3 RESULTS/INTERVENTIONS

The review of evidence confirms that PPCs are a major contributing factor in increasing healthcare costs by lengthening patient hospital stays and increasing morbidity and mortality risk. While several interventions can be used to prevent PPCs, this study examined the effectiveness of pre-op IS education, the use of IS postoperatively, early mobilization, directed cough and deep breathing, and IMT. The results revealed that while some studies concluded that IS, cough and deep breathing exercises, early mobilization, and IMT are effective in preventing postoperative complications, other studies listed here contradict this conclusion. In addition, the review of new research suggested that cumulative interventions (*e.g*., a pulmonary bundle) can be clinically more efficacious in decreasing the incidence of PPCs. Table **1** and Table **2** summarize the details of each study.

### 4 INCENTIVE SPIROMETER (IS) AND PREOPERA-TIVE IS EDUCATION

According to Guimaraes [9], physiotherapy can increase unusually low postoperative functional residual capacity by improving inspiration. IS is an example of physiotherapy, which has been regularly considered a part of the preoperative prophylactic respiratory therapy bundle that aims to prevent and/or treat PPCs. The IS device assists and allows patients to take deep and slow breaths, which increases lung inflation and ultimately promotes optimum gas exchange and adequate lung expansion. As patients establish a routine to continue to use this device at least ten times an hour, it is effective in deterring the development of PPCs [10]. According to Berman **et al*.* [11], by using the IS at the time of inhalation, the flow of air can be measured by the mouthpiece and can assist with overcoming the effects of anesthesia and loosening secretions, which allows for better gas exchange and ventilation. The studies also say that the IS is easy to use and gives patients visual feedback, promoting concordance. In addition, patients can independently use the IS once they are trained. It also provides an effective inspiratory effort and is inexpensive [12]. About 95% of hospitals in the US utilize the IS as a prophylactic measure in patients during both the preoperative and the postoperative period [13].

An experimental study carried out by Khanna [10] tested the efficacy of the use of the IS in postoperative patients who had undergone abdominal or thoracic surgery. This study concluded that using the IS during the preoperative period improves pulmonary function postoperatively. The results showed a marked reduction in the pulmonary function variables of peak expiratory flow rate (PEFR), forced expiratory volume (FEV)1, and FEV6, which were present after surgery. Furthermore, after three and five days postoperatively, IS group patients had superior pulmonary functions when compared to the group carrying out deep-breathing exercises. In addition, the results of these studies were consistent with previous research conducted by Kundra, Vitheeswaran, Nagappa, & Sistla [14], which also concluded that IS is effective in improving postoperative pulmonary complications in abdominal surgery patients. Moreover, this present study also implied compliance was critical for patients in the experimental group, and supervision could be beneficial to ensure an accurate technique and routine use.

As indicated, preventive measures for PPCs should be applied from admission and continued throughout the patient's clinical course. Patients and families should be educated on the potential for postoperative lung complications, the correct use of the IS, and the proper way to breathe deeply and cough. Education during the preoperative period is more effective than when patients are in pain or groggy from sedation during the postoperative period. Patients should be educated on how valuable the IS is and be encouraged to use it at least every 2 hours while awake. Education should be made based on patient preferences. When care providers do not take the time to thoroughly educate patients on the importance of using the IS or watch for a good return demonstration by the patient, the patient ends up with a plastic decoration at their bedside table. Patients need to be encouraged to use it. They must know how to do it correctly: good effort, positioning, frequency, repetition, and coughing. This will achieve the full effect of the IS, such as oxygen saturation and lung expansion, and will prevent the development of basilar crackles.

A randomized study conducted by Bergin **et al*.* [15] on the effect of preoperative IS education (POISE) with a total of 140 participants included instructions for the use of the IS device during the preoperative period (in some cases at home) and the postoperative period. The non-intervention group recorded their postoperative volumes, which were used to determine their baseline volumes. This study's findings revealed that despite no significant difference in the return to baseline volumes between the two groups, the intervention group (POISE) did demonstrate a decrease in the length of hospital stay, postoperative complications, and cost and care. In addition, POISE patients ranked the intervention as helpful and had better outcomes. Preoperative education on IS enhances its postoperative use and reduces patients' complications. Another study conducted by Kundra **et al*.* [14] specifically compared the use of the IS during pre- and postoperative periods in patients undergoing laparoscopic cholecystectomy. This study concluded that lung functions were significantly reduced until discharge in both groups. Still, the overall lung functions were better preserved with the preoperative use of the IS compared to its postoperative use. Additionally, this study pointed out that a lack of efficacy of the IS in previous studies could be due to poor patient compliance. The study by Kundra **et al*.* (14) recommended the prophylactic use of the IS to build up patients' pulmonary reserves as the patients are more motivated and in better physical condition to perform and be carried out under supervision.

In contrast, a search utilizing the Cochrane database for incentive spirometry revealed two relevant meta-analyses, one for upper abdominal surgery and another for pulmonary complications after a coronary artery bypass graft (CABG). Nascimento **et al*.* [16] looked at 12 different studies involving more than 1800 patients and found no apparent benefit of the IS, although they deemed the overall power of the studies to be “low.” The CABG article analyzed seven RCTs and 592 patients; the authors reported that as the quality of evidence is insufficient and unreliable with regards to the effectiveness of the IS in the prevention of PPCs post-CABG, it cannot be concluded whether or not the IS is truly effective. The author also pointed out that most studies had a small number of participants and did not identify substantial variations in the outcome measurements between groups. A systematic review conducted by Rupp, Miley, and Russell-Babin [17] was also evaluated. This study included four systematic reviews and one clinical practice guideline. It concluded that prophylactic use of the IS has not proved more effective than deep breathing or any other single prophylactic technique.

Kokotovic **et al*.* [18] conducted a systematic review and meta-analyses in 2021. They aimed to evaluate the advantages of pulmonary interventions and mobilization postoperatively after abdominal surgery compared with standard protocols to decrease adverse events. Even though the use of postoperative expiratory resistance modalities like continuous positive airway pressure (CPAP), Expiratory Positive Airway Pressure (EPAP), bi-level positive airway pressure (BiPAP), Non-invasive ventilation (NIV) after abdominal surgery might prevent pulmonary complications, there is a need for more data and research to assess the existing evidence. Meta-analyses revealed that compared to standard care, management with high expiratory resistance (CPAP, EPAP, BiPAP, NIV) decreased the chances of respiratory problems. However, they were not able to reach the necessary information size in the sequential trial study.

### 5 EARLY MOBILIZATION

Changing position (*i.e* ., getting into a chair) is advantageous physically and mentally. It should be noted that patients usually have improved lung compliance and the ability to manage respiratory secretions when seated. Also, if patients are always lying down, they tend to feel lightheaded and dizzy when they do get up because of an instant drop in blood pressure. An additional benefit is the strengthening of muscles. The chances of developing acid reflux and breakdown of skin are also decreased when patients are in a seated position. Also, psychologically, the patient will feel better and more alert when not lying in bed 24/7. A systematic research study revealed that several randomized controlled studies had been done to evaluate the effectiveness of deep breathing exercises in postoperative patients. For instance, a randomized controlled study by Silva, Li, and Rickard [19] compared the effects of early and delayed mobilization in postoperative patients. The study concluded that patient mobility, if not started at an early stage, led to increased physiotherapy involvement. However, no substantial difference in the reduction of PPCs between the two groups. In contrast, Haines, Skinner, Berney, and Austin Health POST Study Investigators [20] conducted an observational cohort study examining the association of PPCs with delayed mobilization in postoperative patients. The study revealed a link to an increased incidence of PPC when patients are not mobilized soon after surgery.

### 6 TURN COUGH/DEEP BREATHING

To reduce the risk of pulmonary complications like pneumonia, breathing exercises that increase the effort of inspiration are most useful, as in the study by Silva, Li, and Rickard [19]. They can be useful in postoperative patients with rapid breathing and cough hesitancy. Inactivity and bed rest can lead to volume overload, causing hypostatic pneumonia. However, this adversity can be prevented by directing coughing and deep breathing exercises. Additionally, patients under heavy sedation do not breathe as deeply as they should, leading to pulmonary stasis and infection due to hypoventilation. The correct technique for coughing and deep breathing encourages lung inflation/expansion, opening the airways and moving static secretions, thereby potentially reversing the effects of pulmonary fluid accumulation and avoiding stasis and infection. Postoperative patients who are in pain should be educated on the techniques that will help them, and the importance of early deep breathing exercises should be emphasized; they should be shown how to splint the wound to ease the pain when coughing. Pending more research, recommendations are for deep breathing and coughing postoperatively to decrease the risk of complications. Still, there is insufficient evidence to associate that with reducing postoperative fever. However, an experimental study conducted by Nandi **et al*.* [21] compared the effectiveness of the IS *versus* deep breathing exercises in post-abdominal surgery patients. The results of this study measured the improvement in PEFR over five days. The study concluded that the PEFR was significantly higher among the experimental group compared to the control, thus indicating that IS was more effective than deep breathing.

Arguably, it is important to keep in mind that there were limitations to this study, such as a small sample size, only one outcome measure (PEFR), and the inclusion of abdominal/thoracic surgery patients. We need more research with a bigger sample size to drive practice change. A randomized control study by Lunardi **et al*.* [22] also concluded that deep breathing exercises were ineffective in preventing PPCs. Patients who did deep breathing exercises after abdominal surgery had a higher incidence of PPC compared to the IS group.

### 7 CHEST PHYSIOTHERAPY

In terms of literature evidence on chest physiotherapy, a randomized controlled study conducted by Gbiri, Ajepe, and Akinbo [23] compared the efficacy of IS and chest physiotherapy in 90 patients who had had thoracic/abdominal surgery. A random selection of patients was made, and they were divided into three groups with 30 patients each. All groups received early mobilization. Group 1 was also given selected chest physiotherapy, Group 2 was assigned ISs, and Group 3 was given a combination. All groups had their cardiovascular and pulmonary function checked 24 hours before surgery and during the postoperative period at 24 hours, the third day, and the seventh day. In addition, patients had their blood pressure, mean arterial pressure, pulse pressures, and oxygen hemoglobin saturation monitored for the cardiovascular function check. For the pulmonary function check, patients had forced vital capacity and forced expiratory volume in one second (FEV1) monitored. The results indicated that all clients were 100% compliant with the therapy. Among the experimental groups, there was a significant reduction in cardiovascular and pulmonary complications between 24 hours pre-operatively and 24 hours postoperatively. The study also concluded that chest physiotherapy and the IS were ineffective in reducing postoperative complications, but the combination of these two was clinically more effective. However, participants who received both had a much-improved clinical course and no complications.

Another randomized placebo-controlled superiority trial was conducted by Boden I, **et al*.* [24]. They concluded that a 30-minute physiotherapy session preoperatively in patients scheduled for elective upper abdominal surgery could reduce the risk of developing PPCs and hospital-acquired pneumonia. However, they did mention that more research is required to evaluate the benefits to duration of stay and overall prognosis.

### 8 INSPIRATORY MUSCLE TRAINING

A randomized controlled study by Savci **et al*.* [25] monitored the efficiency of IMT on postoperative CABG surgery patients. For measurements, both groups had their pulmonary function tests (PFTs), 6-minute walk tests (6MWTs), quality of life, and psychosocial status assessed pre-operatively and five days postoperatively. Experimental group patients showed a substantial improvement in inspiratory muscle strength five days postoperatively. This group demonstrated walking a further distance during the 6MWT, improved quality of life as indicated by better sleep quality, a lower anxiety score reflecting better psychosocial status, and—most importantly—a significantly decreased length of stay in the hospital compared to the control group. The conclusion was that IMT positively increased patients' overall pulmonary function post-CABG, resulting in improved and efficient recovery of respiratory muscle strength, functional capacity, short hospital stays, quality of life, and psychosocial status.

In addition, Casali, Pereira, Martinez, de Souza, and Gastaldi [26] conducted a randomized controlled study that analyzed the efficacy of IMT in bariatric surgery patients. They particularly assessed the effects of IMT on PFTs, strength of muscles involved in respiration, and stamina. Their findings concluded that IMT led to an improvement in the strength of inspiratory muscles and stamina. In morbidly obese patients, it also helps with faster recovery of airflows in the respiratory system. Similar studies by Barbalho-Moulim, Miguel, Forti, Campos, and Costa [27], Dettling **et al*.* [28], and Dronkers **et al*.* [29] also concluded that IMT is effective in improving lung expansion. In another study by Valkenet **et al*.* [30], outcomes were similar. They concluded that preoperative exercise therapy with inspiratory muscle training or exercise training before abdominal or cardiac surgeries was associated with reduced hospital length of stay and postoperative complication rates.

Even though numerous studies have confirmed the effectiveness of IMT in reducing PPCs, other studies conclude the opposite. For instance, Kulkarni, Fletcher, McConnell, Poskitt, and Whyman [31] conducted an RCT to assess preoperative IMT on respiratory variables in patients undergoing major abdominal surgery. This study's results indicated no significant reduction in postoperative MIP in the experimental groups concluding that IMT is ineffective in reducing PPCs.

Additionally, a randomized controlled trial was conducted by Lunardi **et al*.* [22] to compare the lung expansion techniques, such as flow IS, deep breathing and volume IS, and their effect on pulmonary volumes, respiratory muscle activation, and the incidence of PPCs in postoperative abdominal surgery patients. Outcomes were collected over a span of 5 days. Results indicated no significant difference in lung volumes and inspiratory muscle activation during lung expansion techniques. Therefore, the authors concluded that the techniques do not prevent PPCs in postoperative abdominal surgery patients. However, they also suggested that more studies must be conducted to confirm their findings and change the standard practice.

Another systematic review by Mans **et al*.* [32] showed that the risk of pulmonary complications can be reduced by half with preoperative IMT. It can lead to the improvement of respiratory muscle function. It should be noted that it did not increase the duration of hospital stay. Going forward, we need more data to ascertain if it decreases the length of stay [32]

### 9 SYNTHESIS OF EVIDENCE

The purpose of this evidence review was to evaluate the effectiveness of each intervention listed above in preventing PPCs. Despite differences in the characteristics of participants and the interventions, it is determined that there is a lack of conclusive evidence to support the effectiveness of a single intervention that would promote lung expansion, thereby reducing and/or preventing PPCs. The main findings indicate that there is mixed research surrounding the use of incentive spirometry and the use of a single prophylactic technique in preventing PPCs in postoperative patients. For instance, some studies state that IS is not recommended alone to prevent postoperative pulmonary complications; IS used with directed coughing, deep breathing techniques, and early mobilization is recommended to avoid PPCs.

Hence, the question is, why do clinicians use them in hospitals? There is no sufficient evidence to say they provide no benefit definitively, so we still use them, but we should have a good understanding of what actual benefit each intervention provides, which may not be much in the case of the IS. While it may “seem like” it does a lot, the whole purpose of obtaining reliable evidence is that what “seems like” it works often does nothing in reality. Arguably, RCTs with a small sample size in a single patient population in a Third World country should not drive changes in practice. Only literature reviews and, preferably, meta-analyses should be used to support the use of ISs. For instance, one of the studies reviewed in the study by Nascimento **et al*.* [16] was limited to 150 patients in an African Hospital in Lilongwe, Malawi. IS was not recommended due to the constrained resource environment. The mortality was 6% in the control group despite early ambulation, coughing, and deep breathing techniques. This is not conclusive evidence, but it is useful information for working in Third World countries. Additionally, it will be challenging to convince a clinician that routinely expanding your lungs while in the healing process is not beneficial.

Research has shown that implementing a respiratory bundle managed by nurses primarily was successful. A study by Mola **et al*.* [33] showed that increasing the use of less invasive respiratory support, such as respiratory bundles, in low birth-weight infants was beneficial. Patients showed significant improvement in oxygenation both immediately after and 15 minutes after breathing exercises were performed in the standing position compared to the sitting position. Even though these interventions are inexpensive and easily promoted, they are a waste of resources if ineffective in improving patient outcomes.

Nonetheless, the cumulative effect of interventions seems optimistic, but more studies need to be conducted. In all studies, the participants ranked education to be an effective intervention. Based on the evidence reviewed for each intervention listed above, research supports a combination of techniques to achieve positive surgical outcomes, as they have a clinically efficacious effect. Postoperatively, it is important to encourage early ambulation soon after surgery to promote lung compliance. Improving blood flow will also help prevent other complications like venous thromboembolism.

Moreover, after a thorough review of the practice and evidence, it seems that applying all interventions as a pulmonary bundle would be more beneficial. To support this further, an observational study conducted by Cassidy, Rosenkranz, McCabe, Rosen, and McAneny [34] examined the efficacy of a multidisciplinary patient care program in reducing PPCs. As a part of this study, Dr. Cassidy and her colleagues designed a program called ICOUGH, which is a multidisciplinary pulmonary care program. It consists of detailed education for patients and caregivers, uniform healthcare provider orders for early ambulation during the postoperative period, and respiratory care. The acronym ICOUGH translates to IS, coughing and deep breathing, oral care (includes brushing teeth and mouthwash use twice a day), understanding (emphasizing patient and caregiver education), getting out of bed (recommend at least three times a day), and **h**ead of the bed elevation. To test the efficacy of this program, the team reviewed the respiratory outcomes before and after the National Surgical Quality Improvement Program (NSQIP) program completion. This program was piloted at Boston University Medical Center and used for general and vascular surgery patients. The program's results were monitored over one year and indicated that the incidence of postoperative pneumonia fell from 2.6% before the implementation of ICOUGH to 1.6% post-ICOUGH program implantation. In addition, the results also showed that after the implementation of ICOUGH, the institution rate of unplanned intubation also decreased, from 2.0% to 1.2%. Thus, if ICOUGH was made a standard practice of care, it could reduce the incidence of postoperative pneumonia and unplanned intubation, saving healthcare organizations a lot of money in the long run.

A similar multicenter observational study was conducted by Jin **et al*.* [35]. It evaluated the occurrence and risk factors associated with developing PPCs in non-cardiac postoperative Chinese patients. It was conducted over six days at four university hospitals and enrolled 1673 participants. Patients were grouped into two different cohorts. Cohort I developed a predictive risk index for PPCs, and Cohort II validated the index. The results showed that 163 (9.7%) patients developed PPCs associated with a more extended hospital stay. The mortality rate among these patients was 1.84%. Cohort I had nine independent risk factors, including tobacco use, pulmonary infection in the last month, use of antibiotics preoperatively, peripheral oxygen saturation preoperatively, surgical site, amount of blood lost, postoperative blood glucose level, albumin level, and ventilation. Cohort II validated these factors. In conclusion, this study successfully developed a risk predictor index for non-cardiac surgery patients. This index can be used as a guide and can estimate the risk of developing PPCs; based on the score, an individualistic respiratory plan can be designed during the perioperative period.

Another study by Wren **et al*.* [36] states that pneumonia is the third most common complication postoperatively. This study also said that having a standardized program has reduced postoperative pneumonia by 40%. If other hospitals adopt this model, it could potentially save $280 million dollars in healthcare costs annually. A CNL, in collaboration with other team members creating a multidisciplinary approach, can pilot a similar program and determine its efficacy by comparing pre- and post-trial results for PPCs.

A number of studies noted there were constraints and limitations to their studies, such as a small number of participants, a lack of diversity, being localized to one area or institution, and needing more variables. For instance, the risk factors for each patient might differ, placing them at a higher risk of developing PPC and thus showing no significant difference in the treatment. In addition, the variation in experimental conditions might not be the same as the subject's daily routine, which can potentially influence and alter the effectiveness of the outcome. With many RCTs with a small sample size (between 20 and 150), it is challenging for the researcher to extract good-quality evidence and make reliable conclusions or statements about the studies. Moreover, factors such as geographic location and the cultural and social diversity of patients involved in the study make it difficult to develop conclusions pertaining to a particular group of individuals or a population. In addition, many studies were methodologically flawed, and the results were differing and not consistent. Out of all the studies reviewed, just two of them clearly explained the process of randomization. Finally, a few studies were performed over seven days or more. Thus, it is possible that a number of environmental and/or extraneous elements may have affected the results throughout this prolonged period.

Understanding the costs and benefits associated with these interventions is crucial for healthcare providers, policymakers, and patients in order to make informed decisions. There are direct and indirect costs associated with postoperative pulmonary complications Direct costs include healthcare expenses such as extended hospital stays, equipment costs, additional training of staff and additional treatments including medications. While indirect costs involve lost productivity due to long recovery times or subsequent disability and the need to maintain updated operating procedures to account for these interventions.

As we have discussed, the use of an incentive spirometer, early mobilization, directed coughing, deep breathing exercises, chest physiotherapy, and inspiratory muscle training (IMT) can lead to improved patient outcomes, shorter hospital stays, and a lower likelihood of long-term complications. Some of these interventions already exist as a part of the post-operative hospital care set for certain patient populations and would, therefore add bias to any cost analyses done that include them. These benefits are significant for patient improvement and satisfaction, even if they don't translate into cost savings. Going forward, detailed cost-effectiveness studies on specific interventions or examining the long-term economic impact of these interventions are needed.

## CONCLUSION

Unfortunately, it is not uncommon for patients to develop PPCs who have undergone surgery, particularly abdominal surgery. Upper abdominal surgeries often lead to rapid shallow breathing, and as a result of this, patients have decreased lung volumes. PPCs can easily increase medical expenditure, translating into longer hospital stays, and more treatments incur more costs. However, the expense can be controlled by decreasing the risk and severity of complications by utilizing therapeutic maneuvers that increase lung volume. The result of the review suggests that cumulative interventions that increase lung expansion during the pre-and postoperative periods might be a solution to prevent PPCs as opposed to a single intervention. More hospitals adopting multi-intervention programs to prevent PPCs can improve patient care and lower mortality, morbidity, and healthcare costs. However, it is essential to conduct RCTs on the efficacy of these interventions individually and combine them with other therapies to find the optimal way to produce the maximum good for the patient.

## Figures and Tables

**Fig. (1):**
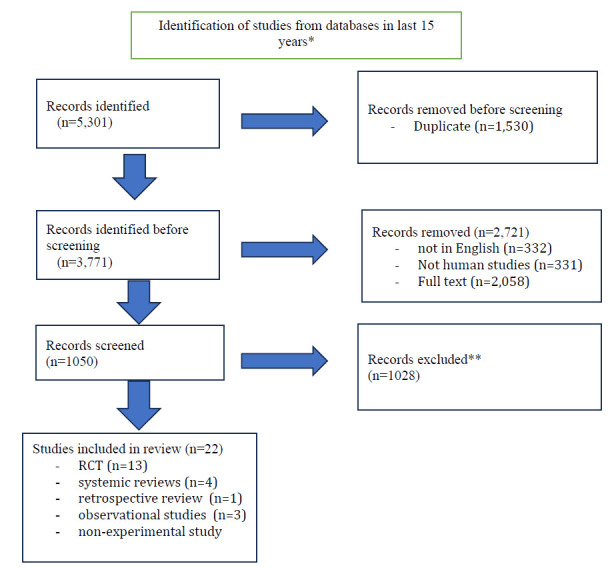
The literature search follows diagram.

**Table 1:** Evidence table template for quantitative research studies

Author, Year/Refs	Study Objective/Intervention or Exposures Compared	Design	Sample (N)	Outcomes Studied	Results	*Quality
Barbalho-Moulim *et al*., 2011 [27]	To evaluate if preoperative inspiratory muscle training can decrease the impact of surgical trauma on the strength of respiratory muscles, diaphragmatic excursions, and lung volumes in obese women having open bariatric surgery.	Randomized controlled trial	32 obese women undergoing elective open bariatric surgery	Respiratory muscle strength (maximal static respiratory pressure - maximal inspiratory pressure and maximal expiratory pressure), lung volume, and diaphragmatic excursion.	Substantial improvement in the maximal inspiratory pressure in the inspiratory muscle training group. Lung volume and diaphragmatic excursion did not increase significantly.	IB
Bergin *et al*., 2014 15]	To examine the results of preoperative incentive spirometry (IS) education (POISE) on postoperative outcomes for knee and hip total joint replacement patients.	Randomized control Trial	N= 140 Group 1 had 50 participants who received the preoperative EDUCATION (POISE) intervention; Group 2 had 56 participants who did not receive any intervention. 34 participants dropped out of the study. Total participants included in study were 106.	Postoperative VOLUMES were recorded for both groups.	No substantial difference between groups in regard to reaching their baseline volume. However, POISE participants had fewer postoperative complications, hospital days, and charges. POISE patients ranked the intervention as helpful.	IB
Casali *et al*., 2011 [26]	To evaluate the effect of inspiratory muscle training (IMT) on pulmonary function, respiratory muscle strength, and endurance in morbidly obese patients undergoing bariatric surgery.	Randomized controlled trial	30 participants were included in the study, and all patients had undergone bariatric surgery	For outcome measurement, the following variables were measured: Forced vital capacity (FVC), forced expiratory volume (FEV), Peak expiratory flow rate (PEFR), maximum static respiratory pressure, and respiratory muscle endurance.	For the results, spirometry measurements, maximum static respiratory pressure, and respiratory muscle endurance were obtained on days 2, 7, 14, and 30 of the post-op period. The data analysis of these measurements indicated that IMT not only increases MIP during the post-op period by 13% (measured on day 30 of post-op) compared to pre-op, but it also increases endurance as well as enables early recovery of spirometry variables such as FEV_1_, PEF, and FEF_25-75%._	IB
Cassidy *et al*., 2013 [33]	To plan, implement, and evaluate the effects of a group of interventions to decrease postoperative pulmonary complications	Observational Study – one hospital	250 patients who underwent general or vascular surgery before and after trial	NSQIP reports were reviewed for incidence and risk-adjusted ratios of postoperative pneumonia and unplanned intubation during one-year pre and post-implementation of the ICOUGH program	Pre-implementation of ICOUGH, according to the NSQIP report, Boston University Medical Center had a 2.6% incidence of postoperative pneumonia and 2.0% of unplanned intubation. Post implementation of ICOUGH, the incidence of post-op complications fell to 1.6%, and the unplanned intubation rate dropped to 1.2%.	IIB
Dettling *et al*., 2013 [28]	To assess the feasibility and initial effect of preoperative inspiratory muscle training (IMT) on the occurrence of pneumonia in patients having oesophagectomy.	Non-randomized controlled trial	39 patients scheduled for oesophagectomy	Incidence of pneumonia postoperatively and duration of hospital stay. Inspiratory muscle strength was measured with a hand-held respiratory pressure meter (Micro Medical RPM; PT Medical, Leek, the Netherlands). Inspiratory muscle endurance was assessed with the incremental threshold loading device (Martyn *et al*., 1987)	Although this pilot study showed no significant differences in the incidence of pneumonia in patients undergoing oesophagectomy, the results show a beneficial effect on respiratory function.	IIB
do Nascimento *et al*., 2013 [16]	To evaluate the benefit of incentive spirometry (IS), compared to no therapy, deep breathing exercises (DBE), or physiotherapy on respiratory complications postoperatively and mortality in adults having upper abdominal surgery.	Systematic review	12 studies with a total of 1834 participants	Occurrence of clinical complications, respiratory failure, and pulmonary complications	The results from participants who received IS were similar to those who received either no treatment, DBE, or physiotherapy in the meta‐analyses for clinical, respiratory, and pulmonary complications. No evidence has been found, that IS, helps in the prevention of PPCs.	IIIB
Dronkers *et al*., 2008 [29]	To investigate the feasibility and effects of preoperative inspiratory muscle training on the occurrence of atelectasis in high-risk pulmonary patients scheduled for elective abdominal aortic aneurysm surgery.	Randomized controlled study	40 participants. Controlled Group – 20 participants. Experimental Group – 20 participants;	Postoperative pulmonary complications (atelectasis); Postoperative respiratory function determined by MIP, inspiratory muscle endurance, and inspiratory vital capacity (25).	The median duration of atelectasis was 0 days in the intervention group and 1.5 days in the control group. Mean postoperative inspiratory pressure was 10% higher in the intervention group.	IB
Ferguson *et al*., 2011 [7]	To identify factors associated with postoperative pulmonary complications in patients who have undergone esophagectomy and to develop a scoring system to identify the relative risk of PPCs.	A retrospective study from 1980 to 2009	516 patients were included in the study	Outcome variables measured included age, forced expiratory volume in 1 second, diffusion capacity of the lung for carbon monoxide, performance status, serum creatinine, smoking status, and transthoracic resection.	A multivariable logistic regression analysis was used in this study to identify predictors of complications. A scoring system was developed to predict pulmonary complications with an accuracy of 70.8%	IIC
Gbiri *et al*., 2016 [23]	To compare the efficacy of selected chest physiotherapy and incentive spirometry in improving cardiovascular and pulmonary functions and preventing adverse events in individuals who had thoracic and/or abdominal surgery.	Randomized control Trial	N= 90 (41 males and 49 females) patients scheduled for elective thoracic and/or abdominal surgery	Systolic Blood Pressure (SBP), Diastolic Blood Pressure (DBP), Mean Arterial Pressure (MAP) and Pulse Pressure (PP) using a mercury sphygmomanometer and a 3M Littmann stethoscope. Forced Vital Capacity (FVC) and the Forced Expiratory Volume in one second (FEV1) with the use of a spirometer (Micro Direct Spirometer MS01).	Either selected chest physiotherapy techniques or incentive spirometry is useful in improving cardiovascular and pulmonary function and preventing complications in individuals who had thoracic and/or abdominal surgery, but the combination of both techniques is clinically more efficacious.	IB
Haines *et al*., 2013 [20]	To measure the incidence, risk factors of PPCs, and barriers to physiotherapy mobilization in patients undergoing abdominal surgery.	An observational cohort study	72 patients undergoing abdominal surgery	Incidence and risk factors for PPCs were measured. Risk factors included; type of incision and time to mobilize away from the bed. Barriers to mobilization and length of stay were also measured.	39% of patients developed PPCs. Results were based on 95% CI 1.2 to 8.0 and indicated that patients were three times more likely to develop PPC with a lack of mobility for each post-op day. On the first post-op day, 52% of patients had variable factors limiting their mobilization. The common barrier was hypotension, although the criteria were not well defined. PPC increased the median length of stay from 13 days to 16. P = 0.046.	IIB
Jin *et al*., 2015 [34]	To examine the occurrence of postoperative pulmonary complications (PPCs) in non-cardiac Chinese patients undergoing surgery and to establish a brief predictive risk index.	Observational Study – Multicenter	1673 participants The cohort was divided into 2 cohorts. Cohort 1, 902 participants were assigned to develop a predictive risk index of PPCs. Cohort II, 771 participants, validated the index.	The risk index was created based on the following data collected from the patients; demographics (such as age, gender, physical status, height & weight, smoking status, alcohol use, and chronic comorbid disease); preoperative variables: respiratory infection in the last month, antibiotic use, lab, anesthetic technique, *etc*.);	1673 patients participated. PPCs were observed in 163 patients (9.7%) who demonstrated prolonged hospital length of stay (LOS). The mortality was 1.84% in patients with PPCs and 0.07% in those without. Logistic Cohort I had nine independent risk factors, including tobacco use, pulmonary infection in the last month, use of antibiotics preoperatively, peripheral oxygen saturation preoperatively, surgical site, amount of blood lost, postoperative blood glucose level, albumin level, and ventilation.	IIB
				postoperative variables: clinical course and laboratory outcomes in 2 hours after surgery. In addition, the occurrence of PPCs, the postoperative length of stay, and the postoperative mortality rate were measured to develop the risk index.	The study was confirmed within cohort II with an area under the receiver operating characteristic curve of 0.90 (95% CI 0.86 to 0.94).	
Khanna, D. S. 2013 [10]	To assess the efficiency of incentive spirometer use in enhancing pulmonary function in patients undergoing upper abdominal surgery.	Randomized control Trial	60 patients had abdominal surgical procedures. Group 1 had 30 patients who used the incentive spirometer, whereas Group 2 also had 30 patients, but this was a control group, and these patients did breathing exercises.	The spirometric values of dependent variables such as post operatively forced vital capacity (FVC) and forced expiratory volume at 1 second (FEV1) Peak expiratory flow rate (PEFR) was measured one day before surgery, three days after surgery, and five days after surgery	For this specific study and data analysis, the significance level was set at p< 0.05 and the confidence limit at 95%. The difference in the value of PEFR a day prior to surgery between the groups was minimal. However, the difference in the value of PEFR three-day and five-day postsurgery between the two groups was significant. In addition, identical results were found for FEV1 and FEV6, where the value difference between the groups was substantial 3 and 5 days post-op.	IB
Kulkarni *et al*., 2010 [31]	This pilot study aimed to evaluate the effect of preoperative inspiratory muscle training (IMT) on respiratory variables in patients having major abdominal surgery.	Randomized controlled trial	80 participants Control Group: 20 participants; Deep breathing exercise: 20 participants; Incentive spirometry: 20 participants; Specific IMT: 20 participants	Length of stay of patients, Respiratory muscle strength (maximum inspiratory [MIP] and expiratory [MEP] mouth pressure), time in HDU/ITU postoperatively, rate of respiration, ventilator time, and oxygen saturation readings at a specific time postoperatively off oxygen, proven respiratory infection (positive sputum culture) and other pulmonary complications	MIP was not elevated from baseline to preoperative assessments in groups A, B, and C. In group D, MIP went up from 51.5 cmH2O (median) pre-training to 68.5 cmH2O (median) post-training preoperatively (*P* < 0.01). Postoperatively, groups A, B, and C decreased in MIP from baseline (*P* < 0.01, *P* < 0.01) and *P* = 0.06, respectively). No substantial decrease in postoperative MIP was noted in group D (*P* = 0.36).	IB
Kundra *et al*., 2010 [14]	This study was constructed to review the efficacy of preoperative and postoperative IS on pulmonary function after laparoscopic cholecystectomy in 50 healthy adults	Randomized control trial	50 Participants Control Group: 25 participants; Intervention Group: 25 participants;	The length of stay, Forced vital capacity (FVC), forced expiratory capacity (FEV1), and peak expiratory flow rate (PEFR) were recorded using the spirobank (25) respirometer (Micro Medicals), and pulmonary complications (infection, positive sputum cultures, CXR indicating pneumonia or atelectasis)	Significant improvement in lung functions was seen after preoperative incentive spirometry (group PR), *P*<0.05. The lung functions were significantly reduced until discharge in both groups. Furthermore, pulmonary functions were markedly well preserved in group PR when compared with group PO; *P*<0.05.	IC
Lunardi *et al*., 2015 [22]	To compare the effects of lung expansion techniques (LET) on pulmonary volume, respiratory muscle activation, and PPC incidence in patients undergoing abdominal surgery.	Randomized controlled trial	137 patients Group One was a control group with 35 patients, Group Two did flow incentive spirometry and had 33 patients, and Group Three did deep breathing exercises. Again, they had 35 patients; lastly, Group Four had 34 patients and did volume incentive spirometry.	Each intervention was performed three times a day for 5 consecutive days. Incidence of pneumonia, atelectasis, or severe hypoxemia was measured in these patients until discharge (which would indicate PPCs). In addition, lung volume and inspiratory muscular activation were measured before and 3 days after surgery.	The results indicated that the incidence of PPC was higher among the deep breathing group (P<.05). Howev- er, postoperatively, no significant change was noted in the lung volume and inspiratory muscular activation during the lung expan sion technique (P>05). Fourteen patients, or 11.2%, developed PPCs.	1B
Nandi *et al*., 2015 [21]	To assess the effectiveness of an incentive spirometer in enhancing the peak expiratory flow rate (PEFR) in abdominal surgery patients.	Randomized controlled trial	40 participants were included in the study. Group A – control group had 20 patients, and patients did diaphragmatic breathing exercises (control) Group B – experimental group had 20 patients, and these patients did breathing exercise as well as IS.	PEFR was measured in both groups on day 1 and day 5.	Outcomes indicated that the experimental group showed significantly higher PEFR compared to the control group. The mean value difference of PEFR between the two groups on day 1 and day 5 was 24.00 and 41.25, with a p-value of 0.021.	IC
Rupp *et al*., 2013 [17]	Compare Use of IS on upper Abdominal surgery *vs*. physiotherapy on control of PPCs	Systematic Review	Use of IS on upper Abdominal surgery(n=35) *vs* physiotherapy(n=35	The use of Incentive spirometry and chest physical therapy is effective in the control of PPCs. It was not clear, although, which of the two was more effective Need for more study	The results of this analysis showed that incentive spirometry and chest physical therapy are more effective than the control in preventing the postoperative pulmonary complications of atelectasis and pneumonia following upper abdominal surgery. However, both interventions usually included positioning and mobilization, and no attempt has been made to determine which mechanisms have more efficacy.	IIB
Savci *et al*., 2011 [25]	To assess the benefits of inspiratory muscle training (IMT) on functional capacity, postoperative respiratory muscle strength, psychosocial status, and quality of life in patients undergoing coronary artery bypass graft (CABG) surgery.	Randomized controlled trial	44 patients underwent CABG surgery and were randomly placed into one of the two groups.	Mean inspiratory muscle strength, functional capacity, intensive care unit stay, quality of life, and psychosocial status.	IMT resulted in an overall improvement in the following categories: Inspiratory muscle strength, functional capacity, intensive care unit stay, quality of life, and psychosocial status after CABG.	IB
Silva *et al*., 2013 [19]	To examine whether adding deep breathing exercises in physiotherapy-directed early mobilization reduces PPCs in patients treated once a day after abdominal surgery. To compare post-op outcomes of early and delayed mobilization.	Cluster Randomized controlled trial	86 patients; Three groups: early mobilization (Group A), early mobilization and breathing exercises (Group B), delayed mobilization (mobilized from the third postoperative day), and breathing exercises (Group C).	PPCs and postoperative outcomes [total number of days until discharge from physiotherapy, physiotherapy input, and length of stay (LOS)].	No substantial difference in PPCs between Groups A and B. The LOS for Group A {mean 10.7 [standard deviation (SD) 5.0] days} was markedly decreased than the LOS for Groups B [mean 16.7 (SD 9.7) days] and C [mean 15.2 (SD 9.8) days; P = 0.0^36^]. The most significant difference was between Groups A and B (mean difference −5.93, 95% confidence interval −10.22 to −1.65; P = 0.008). Group C had less number of smokers (26%) and patients having chronic obstructive pulmonary disease (0%) compared with Group B (53% and 14%, respectively). This may have contributed to fewer PPCs in Group C, but the change was not significant. Despite Group C having less number of PPCs and limited physiotherapy input, the number of days until discharge from physiotherapy and LOS was similar to Group B.	1B
Valkenet *et al*., 2011 [30]	To evaluate the current evidence on the effects of preoperative exercise therapy on postoperative complication rate and length of hospital stay in patients waiting invasive surgery.	Systematic review	12 studies of patients undergoing joint replacement, cardiac or abdominal surgery	Length of hospital stay and complication rates	Preoperative exercise therapy can be effective in reducing postoperative complication rates and duration of hospital stay after cardiac or abdominal surgery	IIIB
Kokotovic *et al*., 2021 [18]	To evaluate if respiratory interventions postoperatively and mobilization interventions compared with standard care can decrease postoperative complications after abdominal surgery	Systematic review	25 studies containing 2068 patients	Pulmonary complications. Postoperative respiratory protocols included expiratory resistance modalities (CPAP, EPAP, BiPAP, NIV), patient-operated ventilation modalities (spirometry, PEP), assisted inspiratory flow modalities (IPPB, IPAP), and structured breathing exercises.	Utilization of postoperative expiratory resistance modalities (CPAP, EPAP, BiPAP, NIV) after abdominal surgery can potentially decrease pulmonary complications	III B
Boden *et al*., 2018 [24]	To evaluate the benefits of a preoperative physiotherapy session to reduce postoperative pulmonary complications (PPCs) in patients undergoing upper abdominal surgery.	Randomized placebo-controlled superiority trial	441 adult patients age 18 years or older who were within six weeks of elective invasive upper abdominal surgery were randomly selected to get an information booklet (n=219; control) or preoperative physiotherapy (n=222; intervention) and followed for 12 months. 432 completed the trial.	The primary outcome was noted to be PPC in a span of 14 postoperative hospital days assessed daily. Secondary outcomes included duration of hospital stay, hospital-acquired pneumonia, hospital costs, and utilization of critical care services.	A 30-minute physiotherapy session preoperatively in patients scheduled for elective upper abdominal surgery can reduce half the risk of developing PPCs and hospital-acquired pneumonia.	IB

* Rating quality of the study (36)

Level:

I: Evidence from the experimental study, RCTS, or meta-analysis of RCTs

II: Evidence from the quasi-experimental study

III: Evidence obtained from a non-experimental study, qualitative study, or meta-synthesis

Quality Rating Scheme

A: High–consistent results with the sufficient sample, adequate control, and definitive conclusions; consistent recommendations based on an extensive literature review that includes thoughtful reference to the scientific literature

B: Good – reasonably consistent results; sufficient samples, some controls, with fairly definitive conclusions; reasonably consistent recommendations based on the fairly comprehensive literature review that includes some reference to scientific evidence

C: Low/major flaws – Little evidence with inconsistent results; insufficient sample size; conclusions cannot be drawn

**Table 2:** Critique template for quantitative research studies

Author, Year/Refs	Study Objective/Intervention or Exposures Compared	Strengths	Weaknesses
Barbalho-Moulim *et al*., 2011 [27]	To evaluate if preoperative inspiratory muscle training can decrease the effect of surgical trauma on respiratory muscle strength in the lung volumes and diaphragmatic excursion in obese women undergoing open bariatric surgery.	Design: Experimental design – particularly random assignment of subjects Sampling: Clearly defined inclusion/exclusion criteria. Methods: Conceptualization was well developed. The study protocol was clearly articulated. Results: Thoroughly displayed results in text and table.	Sampling: Small sample size (strong likelihood of Type II error) and a convenience sample Analysis: No power analysis External Validity: Limited generalizability
Bergin, *et al*., 2014 [15]	To compare the effect of giving oxygen by mask, deep breathing exercises, and positive expiratory pressure on the control of PPCs	Design: Experimental design – particularly random assignment of subjects, both the control and test subjects were well monitored. Sampling: Clearly defined inclusion/exclusion criteria. Methods: Conceptualization was well developed. The study protocol was clearly articulated. Results: Thoroughly displayed in text and table. Interpretation was okay	Sampling: Small sample size (strong likelihood of Type II error) and convenience sample. Research is warranted with a bigger sample size. Methods: Both compliance and pain medication use could alter VOLUMES achieved by patients. Patients discharged who had not yet achieved a postoperative RETURN to baseline volume during hospitalization were also not followed. Analysis: No power analysis External Validity: Limited generalizability Results could not be quantified Difficult with the selection of subjects
Casali *et al*., 2011 [26]	To evaluate the effect of inspiratory muscle training (IMT) on pulmonary function, respiratory muscle strength, and endurance in morbidly obese patients undergoing bariatric surgery.	Design: Experimental design – particularly random assignment of subjects Sampling: Clearly defined inclusion/exclusion criteria. Methods: Conceptualization was well developed. The study protocol was clearly articulated. Results: Thoroughly displayed in text and table.	Sampling: Small sample size (strong likelihood of Type II error) and a convenience sample Methods: More variables could have been evaluated. Analysis: No power analysis External Validity: Limited generalizability
Cassidy *et al*., 2013 [33]	To plan, implement, and evaluate the effects of a group of interventions to decrease postoperative pulmonary complications	Design: Before and after the trial program Sampling: Clearly defined inclusion/exclusion criteria. Methods: Conceptualization was well developed. Study protocol was clearly articulated. Results: Thoroughly displayed in text and table.	Analysis: No power analysis External Validity: do not yet have data to confirm the sustained success of the I COUGH program over a prolonged period of time, although early trends are encouraging.
Dettling *et al*., 2013 [28]	To assess the feasibility and initial effect of preoperative inspiratory muscle training (IMT) on the occurrence of pneumonia in patients having oesophagectomy.	Design: Experimental design – particularly random assignment of subjects Sampling: Clearly defined inclusion/exclusion criteria. Methods: Conceptualization was well developed. The study protocol was clearly articulated. Results: Thoroughly displayed in text and table.	Sampling: Small sample size (strong likelihood of Type II error) and a convenience sample Design: The study design of this pilot study does not allow us to draw conclusions with respect to the effectiveness of IMT. The assignment was not random but based on the distance to the hospital. Methods: The study was not powered to show statistically significant differences in the outcome measures but was set up as a pragmatic preliminary trial including a homogeneous cohort of 90 consecutive patients and only looked at patients with pneumonia for PPCs. Analysis: No power analysis External Validity: Limited generalizability
do Nascimeno *et al*., 2013 [16]	To evaluate the benefit of incentive spirometry (IS), compared to no therapy or deep breathing exercises (DBE), or physiotherapy, on respiratory complications postoperatively and mortality in adults having upper abdominal surgery.	Design: Systematic review of 12 studies, all RCTs. Meta-analysis Sampling: Clearly defined inclusion/exclusion criteria. Methods: Using instruments with proven validity and reliability. The study protocol was clearly articulated. Results: Thoroughly discussed in the text and tables.	Sampling: 12 studies and most studies had a small sample and lots of biases, unclear methodology. Analysis: meta-analysis External Validity: The majority of the studies in this review were old and carried out before there was understanding with regard to the internal validity of RCTs. In addition, the included studies had no standardized outcomes resulting in challenges with the performance of a meta-analysis.
Dronkers *et al*., 2008 [29]	To investigate the feasibility and effects of preoperative inspiratory muscle training on the occurrence of atelectasis in high-risk pulmonary patients scheduled for elective abdominal aortic aneurysm surgery.	Design: Experimental design – particularly random assignment of subjects Methods: Conceptualization was well developed. The study protocol was clearly articulated. Results: Thoroughly explained in text and table.	Sampling: Small sample size (strong likelihood of Type II error) and convenience sample. No clearly defined inclusion/exclusion criteria. Analysis: No power analysis External Validity: Limited generalizability
Ferguson *et al*., 2011 [7]	To identify factors that are associated with postoperative pulmonary complications in patients who have undergone esophagectomy and to develop a scoring system to identify the relative risk of PPCs.	Design: Retrospective review The study protocol was clearly articulated. Results: Thoroughly displayed in text and table.	Sampling: Small sample size (strong likelihood of Type II error) and convenience sample. Design: This was a single-center study encompassing all attendant drawbacks of such an effort. Methods: Not well conceptualized. Analysis: Univariate analysis; data collection was incomplete, mainly because not all variables were measured in each patient, and this required multiple imputation techniques to complete the data set before analysis. External Validity: Limited generalizability
Gbiri *et al*. 2016 [23]	Use of chest physiotherapy in the prevention of PPCs	Design: Experimental design – particularly random assignment of subjects. Both the control and test subjects were well monitored. Sampling: Clearly defined inclusion/exclusion criteria. Methods: Conceptualization was well developed. The study protocol was clearly articulated. Results: Detailed discussion in text and tables. Interpretation was okay	Sampling: Small sample size (strong likelihood of Type II error) and a convenience sample Methods: Difficult with the selection of subjects Analysis: No power analysis External Validity: Limited generalizability; more studies are recommended to confirm results.
Haines *et al*., 2013 [20]	To measure the incidence, risk factors of PPCs, and barriers to physiotherapy mobilization in patients undergoing abdominal surgery.	Design: An observational cohort study Sampling: Clearly defined inclusion/exclusion criteria. Methods: Conceptualization was well developed. The study protocol was clearly articulated. Results: Thorough discussion in text and tables.	Design: single-center design, high-risk patients who required ICU admission. Methods: Results were collected over 7 days and only when the patient was with a physiotherapist. Any mobilization that occurred outside of physiotherapist treatment did not get recorded. Meaning some patients could have been missed. Analysis: No power analysis External Validity: high-risk patients and highly selected nature could also affect the generalizability of the study.
Jin *et al*., 2015 [34]	To examine the occurrence of postoperative pulmonary complications (PPCs) in non-cardiac Chinese patients undergoing surgery and to establish a brief predictive risk index.	Sampling: Clearly defined inclusion/exclusion criteria. Methods: Conceptualization was well developed. The study protocol was clearly articulated. Results: Thoroughly discussed and displayed in text and table.	Sampling: Only four hospitals participated, which may have resulted in some bias—a small sample. More extensive studies are needed. Methods: Study period of 7 days. Enrollment was carried out in August for all patients. Another limitation is that. Englesbe **et al*.* displayed a remarkable seasonal variability in surgical morbidity and mortality. They found a significant increase in surgical mortality in July, largely due to onboarding interns. However, Ehlert and his colleagues argued against the “July Phenomenon” in a bigger population. In the majority of the hospitals in China, the influx of interns is notably high in July. They mostly do the paperwork as opposed to providing direct patient care. As a result, their effects on the outcomes are limited. More research is needed to assess the effects of seasonal variability on morbidity and mortality in surgical patients. Analysis: No power analysis External Validity: Limited generalizability
Khanna, D. S., 2013 [10]	To assess the efficiency of incentive spirometer use in enhancing pulmonary function in patients undergoing upper abdominal surgery.	Design: Experimental design – particularly random assignment of subjects Sampling: Clearly defined inclusion/exclusion criteria. Methods: Conceptualization was well developed. The study protocol was clearly articulated. Results: Thorough discussion in text and tables.	Sampling: Small sample size (strong likelihood of Type II error) and convenience sample Methods: Only three aspects of pulmonary function, PEFR, FEV1, and FEV6, are taken in this study. External Validity: Limited generalizability
Kulkarni *et al*., 2010 [31]	The aim of this pilot study was to evaluate the effect of preoperative inspiratory muscle training (IMT) on respiratory variables in patients having major abdominal surgery.	Design: Experimental design – particularly random assignment of subjects Sampling: To allow the results to be inclusive of a wider age group, the inclusion and exclusion criteria were purposely made less stringent in patients having abdominal surgery, Methods: Conceptualization was well developed. Study protocol was clearly articulated. Results: Detailed discussion in text and tables.	Sampling: Small sample size (strong likelihood of Type II error) and a convenience sample Methods: Patients not likely to wait for 2 weeks to have the surgery were excluded as a result of the strict exclusion criteria. In addition, many patients in the queue for intra-abdominal cancer surgery were not part of the study and, as a result, might not have an opportunity in the future to improve their breathing before surgery. Thereby, it might be essential to have a flexible training period to extend opportunities to patients in need of urgent surgery. Analysis: No power analysis External Validity: Limited generalizability
Kundra *et al*., 2010 [14]	This study was constructed to review the efficacy of preoperative and postoperative IS on pulmonary function after laparoscopic cholecystectomy in 50 healthy adults.	Design: Experimental design – particularly random assignment of subjects Sampling: Clearly defined inclusion/exclusion criteria. Methods: Conceptualization was well developed. Using instruments with proven validity and reliability. The study protocol was clearly articulated. Results: Detailed discussion in text and table	Sampling: Small sample size (strong likelihood of Type II error) and a convenience sample Analysis: No power analysis External Validity: Limited generalizability
Lunardi *et al*., 2015 [22]	To compare the effects of lung expansion techniques (LET) on pulmonary volume, respiratory muscle activation, and PPC incidence in patients undergoing abdominal surgery.	Design: Experimental design – particularly random assignment of subjects Sampling: Clearly defined inclusion/exclusion criteria. All groups were homogenous for age, sex, BMI, lung function, and thoracoabdominal mechanics. Methods: Conceptualization was well developed. Using instruments with proven validity and reliability. The study protocol was clearly articulated. Results: Thoroughly displayed in text and table.	Methods: The only predictors of PPC were Higher American Society of Anesthesiologists scores and surgery duration. Analysis: No power analysis External Validity: Limited generalizability
Nandi *et al*., 2015 [21]	To assess the effectiveness of incentive spirometer in enhancing the peak expiratory flow rate (PEFR) in abdominal surgery patients.	Design: Experimental design – particularly random assignment of subjects Sampling: Clearly defined inclusion/exclusion criteria. Methods: Conceptualization was well developed. The study protocol was clearly articulated. Results: Thoroughly discussed in the text and table.	Sampling: Small sample size (strong likelihood of Type II error) and a convenience sample Methods: Only one outcome measure, *i.e* ., PEFR. Only abdominal surgery patients are included. Analysis: No power analysis External Validity: Limited generalizability
Rupp *et al*., 2013 [17]	Compare the Use of IS on upper Abdominal surgery *vs*. physiotherapy on control of PPCs	Methods: Having a multidisciplinary team to review and discuss, each article allowed all results, including those found within the individual studies contributing to the systematic reviews, to be compared from many viewpoints of clinical meaningfulness. Results: Detailed discussion in text and tables. This review of the literature also lends itself to disseminating important clinical information throughout the nursing community, having been performed to enhance nursing practice.	Design: Systematic review review of the literature was the small number of citations available within journals of nursing literature. The absence of any nursing literature on this subject excludes the perspective of the discipline, that is the key user of the incentive spirometry device. Methods: A lack of specific inclusion/exclusion criteria for both patient populations and evaluation of outcomes in the articles reviewed. For example, although some of the RCTs included in a systematic review may have measured the incidence of atelectasis postoperatively, others may have used measures of functional lung capacity postoperatively to indicate outcomes. These inconsistencies between measured outcomes detracted from the strength of even the strongest systematic reviews. In addition, the broad spectrum of surgical procedures encompassed within the realm of “abdominal/thoracic surgical procedures” increases the difficulty of comparing patient outcomes across different surgical procedures.
Savci *et al*., 2011 [25]	To assess the benefits of inspiratory muscle training (IMT) on functional capacity, postoperative respiratory muscle strength, psychosocial status, and quality of life in patients undergoing coronary artery bypass graft (CABG) surgery.	Design: Experimental design – particularly random assignment of subjects Sampling: Clearly defined inclusion/exclusion criteria. Methods: Conceptualization was well developed. The study protocol was clearly articulated. Results: Thoroughly displayed in text and table.	Sampling: Small sample size (strong likelihood of Type II error) and convenience sample. One limitation of this study is the small sample size. Therefore, the long-term effects of inspiratory muscle training in a wider sample size group of CABG patients should be investigated. Methods: The long-term effects of inspiratory muscle training in patients were not monitored. Patients were trained for only two weeks (5 – 7 days preoperatively, 5 days postoperatively). Although a short-term inspiratory muscle training program was applied, the program could have provided maintenance of respiratory muscle strength and functional capacity. Secondly, patients with low-risk scores were included in this study. Further studies in patients with different risk scores and extended follow-up periods after CABG are warranted to confirm the results. Thirdly, a generic health status questionnaire was used to evaluate the quality of life. The measurement of quality of life using a disease-specific questionnaire may identify areas of improvement. Besides, more contact with the intervention group might have a motivational effect on quality of life and anxiety. It should be investigated. Analysis: No power analysis External Validity: Limited generalizability
Silva *et al*., 2013 [19]	To examine whether adding deep breathing exercises in physiotherapy-directed early mobilization reduces PPCs when patients are treated once daily after abdominal surgery. To compare post-op outcomes of early and delayed mobilization.	Design: Clustered Randomized control trial Results: Thoroughly displayed and discussed in text and tables.	Sampling: Small sample size and convenience sample, Single-center study in a teaching hospital. Design: With cluster randomization, it is unclear whether there was some contamination with patients undergoing surgery at the end of the week and those undergoing surgery at the beginning of the following week. Methods: Despite being blindsided by patient allocation, medical and nursing staff may have encouraged deep breathing exercises because these exercises are routine in postoperative management. In addition, patient adherence to deep breathing exercises and length of time sat out of bed were not measured. Analysis: No power analysis External Validity: Limited generalizability
Valkenet *et al*., 2011 [30]	To summarize the current evidence on the effects of preoperative exercise therapy on postoperative complication rate and length of hospital stay in patients awaiting invasive surgery.	Design: Systematic review that uses more than one randomized control study in the review of literature Sampling: Clearly defined inclusion/exclusion criteria. Methods: The PEDro scale was used to assess the methodological quality of the included studies independently. Statistical pooling was completed when studies were comparable in terms of outcome measures and patient population. Results: Results were separately described if pooling was not possible.	Design: A systematic review Analysis: No power analysis External Validity: Limited generalizability
Kokotovic *et al*., 2021 [18]	To evaluate if respiratory interventions postoperatively and mobilization interventions compared with standard care can decrease postoperative complications after abdominal surgery	Design: Systematic review and meta-analysis. Sampling: Clearly defined inclusion/exclusion criteria. Methods: Using instruments with proven validity and reliability. The study protocol was clearly articulated. Results: Detailed discussion in text and tables.	Sampling: They were not able to reach the necessary information size in the sequential trial study. Methods: Only abdominal surgery patients are included
Boden *et al*., 2018 [24]	To evaluate the benefits of a preoperative physiotherapy session to reduce postoperative pulmonary complications (PPCs) in patients undergoing upper abdominal surgery.	Design: Prospective, pragmatic, multicentre, patient and assessor-blinded, parallel-group, randomized placebo-controlled superiority trial. Sampling: Clearly defined inclusion/exclusion criteria. Methods: Conceptualization was well developed. The study protocol was clearly articulated. Results: Thoroughly discussed in the text and table.	Sampling: Small sample size Methods: The evidence is limited due to weakness in methodology and poor generalizability.

## LIST OF ABBREVIATIONS

PPCs: Postoperative pulmonary complications
IS: incentive spirometer
IMT: inspiratory muscle training
RCTs: randomized controlled trials
PEFR: peak expiratory flow rate
FEV: forced expiratory volume
IMT: inspiratory muscle training

## References

[1] Kazaure H.S., Roman S.A., Sosa J.A. (2012). Association of postdischarge complications with reoperation and mortality in general surgery.. Arch. Surg..

[2] Shander A., Fleisher L.A., Barie P.S., Bigatello L.M., Sladen R.N., Watson C.B. (2011). Clinical and economic burden of postoperative pulmonary complications: Patient safety summit on definition, risk-reducing interventions, and preventive strategies.. Crit. Care Med..

[3] Crema E., Benelli A.G., Silva A.V., Martins A.J., Pastore R., Kujavao G.H., Silva A.A., Santana J.R. (2005). Assessment of pulmonary function in patients before and after laparoscopic and open esophagogastric surgery.. Surg. Endosc..

[4] Levin M.A., McCormick P.J., Lin H.M., Hosseinian L., Fischer G.W. (2014). Low intraoperative tidal volume ventilation with minimal PEEP is associated with increased mortality.. Br. J. Anaesth..

[5] Zaky A.L. (2011). The use of intraoperative positive end expiratory pressure.. J. Anesth. Clin. Res..

[6] Zingg U., Smithers B.M., Gotley D.C., Smith G., Aly A., Clough A., Esterman A.J., Jamieson G.G., Watson D.I. (2011). Factors associated with postoperative pulmonary morbidity after esophagectomy for cancer.. Ann. Surg. Oncol..

[7] Ferguson M.K., Celauro A.D., Prachand V. (2011). Prediction of major pulmonary complications after esophagectomy.. Ann. Thorac. Surg..

[8] T Duarte A., S Machado H. (2016). Postoperative pulmonary complications: An epidemiological, risk factors and prevention review.. J. Anesth. Clin. Res..

[9] Guimarães M.M., El Dib R., Smith A.F., Matos D. (2009). Incentive spirometry for prevention of postoperative pulmonary complications in upper abdominal surgery.. Cochrane Database Syst. Rev..

[10] Khanna S. (2013). Efficacy of incentive spirometer in improving pulmonary functions after upper abdominal surgery.. Indian J. Basic Appl. Med. Res..

[11] Berman A., Kozier B., Berman A. (2012). Kozier & Erb’s fundamentals of nursing: concepts, process, and practice..

[12] Westwood K., Griffin M., Roberts K., Williams M., Yoong K., Digger T. (2007). Incentive spirometry decreases respiratory complications following major abdominal surgery.. Surgeon.

[13] O’Donohue W.J. (1985). National survey of the usage of lung expansion modalities for the prevention and treatment of postoperative atelectasis following abdominal and thoracic surgery.. Chest.

[14] Kundra P., Vitheeswaran M., Nagappa M., Sistla S. (2010). Effect of preoperative and postoperative incentive spirometry on lung functions after laparoscopic cholecystectomy.. Surg. Laparosc. Endosc. Percutan. Tech..

[15] Bergin C., Speroni K.G., Travis T., Bergin J., Sheridan M.J., Kelly K., Daniel M.G. (2014). Effect of preoperative incentive spirometry patient education on patient outcomes in the knee and hip joint replacement population.. J. Perianesth. Nurs..

[16] do Nascimento Junior P., Módolo N.S.P., Andrade S., Guimarães M.M.F., Braz L.G., El Dib R. (2014). Incentive spirometry for prevention of postoperative pulmonary complications in upper abdominal surgery.. Cochrane Libr..

[17] Rupp M., Miley H., Russell-Babin K. (2013). Incentive spirometry in postoperative abdominal/thoracic surgery patients.. AACN Adv. Crit. Care.

[18] Kokotovic D., Berkfors A., Gögenur I., Ekeloef S., Burcharth J. (2021). The effect of postoperative respiratory and mobilization interventions on postoperative complications following abdominal surgery: A systematic review and meta-analysis.. Eur. J. Trauma Emerg. Surg..

[19] Silva Y.R., Li S.K., Rickard M.J.F.X. (2013). Does the addition of deep breathing exercises to physiotherapy-directed early mobilisation alter patient outcomes following high-risk open upper abdominal surgery? Cluster randomised controlled trial.. Physiotherapy.

[20] Haines K.J., Skinner E.H., Berney S. (2013). Association of postoperative pulmonary complications with delayed mobilisation following major abdominal surgery: An observational cohort study.. Physiotherapy.

[21] Nandi B., Mishra S., Yeole U., Gawali P., Adkitte R. (2015). Effectiveness of incentive spirometry in improving peak expiratory flow rate in post abdominal surgery : An experimental study.. J. Med. Thes..

[22] Lunardi A.C., Paisani D.M., Silva C.C.B.M., Cano D.P., Tanaka C., Carvalho C.R.F. (2015). Comparison of lung expansion techniques on thoracoabdominal mechanics and incidence of pulmonary complications after upper abdominal surgery: A randomized and controlled trial.. Chest.

[23] Gbiri C., Ajepe T., Akinbo S. (2016). Efficacy of chest-physiotherapy and incentive-spirometry in improving cardiovascular and pulmonary functional performances in individuals post-thoraco-abdominal surgery: A randomised comtrolled study.. Int. J. Therap. Rehabil. Res..

[24] Boden I., Skinner E.H., Browning L., Reeve J., Anderson L., Hill C., Robertson I.K., Story D., Denehy L. (2018). Preoperative physiotherapy for the prevention of respiratory complications after upper abdominal surgery: Pragmatic, double blinded, multicentre randomised controlled trial.. BMJ.

[25] Savci S., Degirmenci B., Saglam M., Arikan H., Inal-Ince D., Turan H.N., Demircin M. (2011). Short-term effects of inspiratory muscle training in coronary artery bypass graft surgery: A randomized controlled trial.. Scand. Cardiovasc. J..

[26] Casali C.C.C., Pereira A.P.M., Martinez J.A.B., de Souza H.C.D., Gastaldi A.C. (2011). Effects of inspiratory muscle training on muscular and pulmonary function after bariatric surgery in obese patients.. Obes. Surg..

[27] Barbalho-Moulim M.C., Miguel G.P.S., Forti E.M.P., do Amaral Campos F., Costa D. (2011). Effects of preoperative inspiratory muscle training in obese women undergoing open bariatric surgery: respiratory muscle strength, lung volumes, and diaphragmatic excursion.. Clinics.

[28] Dettling D.S., van der Schaaf M., Blom R.L.G.M., Nollet F., Busch O.R.C., van Berge Henegouwen M.I. (2013). Feasibility and effectiveness of pre-operative inspiratory muscle training in patients undergoing oesophagectomy: A pilot study.. Physiother. Res. Int..

[29] Dronkers J., Veldman A., Hoberg E., van der Waal C., van Meeteren N. (2008). Prevention of pulmonary complications after upper abdominal surgery by preoperative intensive inspiratory muscle training: A randomized controlled pilot study.. Clin. Rehabil..

[30] Valkenet K., van de Port I.G.L., Dronkers J.J., de Vries W.R., Lindeman E., Backx F.J.G. (2011). The effects of preoperative exercise therapy on postoperative outcome: A systematic review.. Clin. Rehabil..

[31] Kulkarni S.R., Fletcher E., McConnell A.K., Poskitt K.R., Whyman M.R. (2010). Pre-operative inspiratory muscle training preserves postoperative inspiratory muscle strength following major abdominal surgery : A randomised pilot study.. Ann. R. Coll. Surg. Engl..

[32] Mans C.M., Reeve J.C., Elkins M.R. (2015). Postoperative outcomes following preoperative inspiratory muscle training in patients undergoing cardiothoracic or upper abdominal surgery: A systematic review and meta analysis.. Clin. Rehabil..

[33] Mola S.J., Annibale D.J., Wagner C.L., Hulsey T.C., Taylor S.N. (2015). NICU bedside caregivers sustain process improvement and decrease incidence of bronchopulmonary dysplasia in infants < 30 weeks gestation.. Respir. Care.

[34] Cassidy M.R., Rosenkranz P., McCabe K., Rosen J.E., McAneny D. (2013). I COUGH: Reducing postoperative pulmonary complications with a multidisciplinary patient care program.. JAMA Surg..

[35] Jin Y., Xie G., Wang H., Jin L., Li J., Cheng B., Zhang K., Hoeft A., Fang X. (2015). Incidence and risk factors of postoperative pulmonary complications in noncardiac Chinese patients: A multicenter observational study in university hospitals.. BioMed Res. Int..

[36] Wren S.M., Martin M., Yoon J.K., Bech F. (2010). Postoperative pneumonia-prevention program for the inpatient surgical ward.. J. Am. Coll. Surg..

